# Single-molecule tracking dataset of histone H3 (Hht1) in *Saccharomyces cerevisiae*

**DOI:** 10.1016/j.dib.2023.108925

**Published:** 2023-01-25

**Authors:** Nitesh Kumar Podh, Ayan Das, Partha Dey, Sheetal Paliwal, Gunjan Mehta

**Affiliations:** Laboratory of Chromosome Dynamics and Gene Regulation, Department of Biotechnology, Indian Institute of Technology Hyderabad, Kandi, Sangareddy, Telangana 502285, India

**Keywords:** Yeast, Diffusion coefficient, Residence time, Bound fraction, Jump angles, Jump distances, Chromatin

## Abstract

Single-Molecule Tracking (SMT) is a powerful method to quantify protein dynamics in live cells. Recently, we have established a data analysis pipeline for estimating various biophysical parameters (mean squared displacement, diffusion coefficient, bound fraction, residence time, jump distances, jump angles, and track statistics) from the single-molecule time-lapse movies acquired from yeast *Saccharomyces cerevisiae*. We acquired the time-lapse movies using different time intervals (i.e. 15 ms, 200 ms, and 1000 ms) to extract the diffusion parameters (from 15 ms time interval movies) and residence time (from 200 ms and 1000 ms time interval movies). We tracked the single molecules from these movies using three MATLAB-based software packages (MatlabTrack, TrackIT, DiaTrack (Sojourner, and Spot-On)) to quantify various biophysical parameters. In this article, we have quantified the biophysical parameters of chromatin-bound histone H3 (Hht1), labeled using JF646 HaloTag Ligand (HTL), and shared a few raw time-lapse SMT movies for the same. Histone H3 is a chromatin-bound protein and it serves as a benchmark for the stably bound molecules for the SMT experiments. Hence, this dataset can be used by various researchers to quantify the biophysical parameters of chromatin-bound molecules (Histone H3). Any newly developed tracking software can use this dataset to validate the accuracy of its tracking algorithms.

**Specifications Table**

**Table:** 

Subject	Biophysics
Specific subject area	Single-molecule tracking (SMT) microscopy of histone H3 in live cells of yeast *S. cerevisiae* to quantify diffusion parameters and residence time
Type of data	Raw time-lapse microscopic movies (.tiff) Tracked data files using MATLAB-based software packages (MatlabTrack, TrackIt, DiaTrack, and Sojourner) Graph Figure Image
How the data were acquired	To visualize the single-molecules of histone H3 (Hht1), we fused -HaloTag at the c-terminus of the *HHT1* gene endogenously and sparsely labeled with the JF646 HaloTag ligand (0.005 nM). The cells were placed in a LabTek II imaging chamber, covered with a nutrient agarose pad, and observed under the custom-built HILO (Highly Inclined and Laminated Optical sheet) microscope (100X 1.49 NA TIRF objective; Lasers (OBIS, Coherent): 488 nm (100 mW), 647 nm (100 mW); EMCCD: iXon Ultra 888, Andor Technology). The time-lapse movies were acquired with 15 ms, 200 ms, and 1000 ms time intervals and 15 ms exposure time, with a pixel size of 117 nm. These datasets were tracked using MATLAB-based tracking software.
Data format	Raw Analyzed
Description of data collection	We acquired the time-lapse movies with fast (0 ms interval, 15 ms exposure) and slow (200 and 1000 ms intervals, 15 ms exposure) imaging regimes. The fast-imaging movies were used for quantifying the diffusion parameters (MSD, *D*, bound fraction, jump distances etc), whereas, the slow-imaging movies were used for quantifying the residence time. These datasets were tracked using three MATLAB-based software: MatlabTrack, TrackIt, and Diatrack (Sojourner, Spot-On).
Data source location	*• Institution: National Cancer Institute, National Institutes of Health* *• City/Town/Region: Bethesda* *• Country: USA*
Data accessibility	Repository name: Mendeley Data Data identification number: doi: 10.17632/xkjp4nm33h.2 Direct URL to data: https://data.mendeley.com/datasets/xkjp4nm33h
Related research article	N.K. Podh, A. Das, P. Dey, S. Paliwal, G. Mehta, Single-Molecule Tracking for studying protein dynamics and target-search mechanism in live cells of *S. cerevisiae,* STAR Protoc. 3 (2022) 101900. https://doi.org/10.1016/j.xpro.2022.101900 [1]

## Value of the Data

• Histone H3 is a chromatin-bound protein, hence it serves as a benchmark for stable binding for single-molecule tracking experiments. In this dataset, we have acquired the time-lapse movies for single-molecule tracking of histone H3 using fast- and slow-imaging regimes.
• This dataset can be used as a control for the chromatin-bound molecule by any researcher who wishes to quantify the single-molecule dynamics of any other protein of interest.
• This dataset defines the upper limit of the biophysical parameters that can be quantified using single-molecule tracking, such as diffusion coefficient, residence time, and bound fraction.
• Any new/old tracking software/algorithms can be tested using this dataset to validate their performance and to compare the values of the biophysical parameters quantified using various software (for performance comparison purposes).
• This dataset can be used as a training dataset for developing machine learning-based approaches for single-molecule tracking.

## 1 Objective

In the last decade, single-molecule tracking (SMT) has been extensively used to understand the diffusion dynamics and target-search mechanism of several proteins in the live cells of yeast *Saccharomyces cerevisiae* [2], [3], [4], [5], [6], [7]*.* Any SMT experiment needs control for stably-bound molecules. Histone H3 is a chromatin-bound protein, hence, it can be used as a benchmark for the stably-bound molecule. Here, we have generated a dataset for SMT of histone H3 (time-lapse movies acquired with 15 ms, 200 ms, and 1000 ms time intervals with 15 ms exposure time and 117 nm pixel size). We also tracked these movies with three different tracking software to extract various biophysical parameters such as diffusion coefficient, bound fraction, residence time, jump distances and angles, and target-search parameters. This data article is related to the STAR Protocols article [1], in which the details of the experimental procedure and data analysis pipeline are described. This data article demonstrates the quantification of the biophysical parameters for histone H3. The values shown in this article define the upper limit of those biophysical parameters that can be estimated by SMT.

## 2 Data Description

We fused the *HHT1* gene of *S. cerevisiae* endogenously with -HaloTag using PCR-based homologous recombination. For visualizing nuclear boundaries, we expressed GFP-LacI with NLS (nuclear localization sequence) in the same yeast strain. The log phase cells were treated with 0.005 nM JF646-HaloTag Ligand (HTL) for sparse labeling of the histone H3 molecules. The cells were placed in the LabTek II imaging chamber, covered with a nutrient agarose pad, and observed under the custom-built HILO microscope (100X 1.49 NA TIRF objective; Lasers (OBIS, Coherent): 488 nm (100 mW), 647 nm (100 mW); EMCCD: iXon Ultra 888, Andor Technology). Single-focal plane time-lapse movies were acquired in a 646 nm channel using fast (0 ms interval, 15 ms exposure) and slow (200 and 1000 ms intervals, 15 ms exposure) imaging regimes. For the reference image for nuclear boundaries, we acquired a single-focal plane, and a single time point image in a 488 nm channel (500 ms exposure) after acquiring a time-lapse movie in a 647 nm channel.

**Fig. 1:**
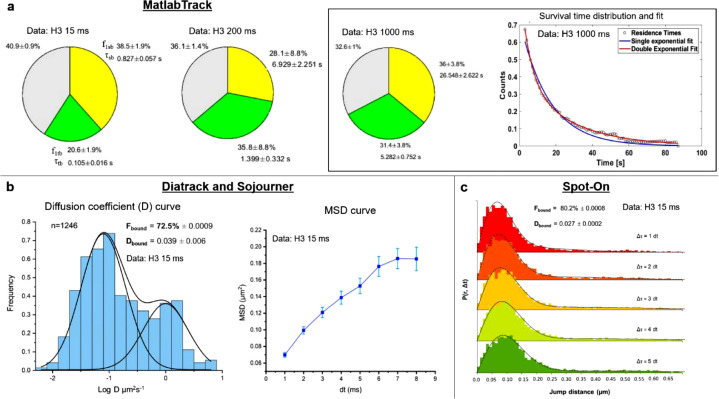
**a)** Fraction of chromatin bound molecules and their residence times, as obtained by MatlabTrack for 15 ms, 200 ms, 1000 ms time interval data. The survival time distribution and fit are shown for 1000 ms data. **b)** Diffusion coefficient distribution and MSD curve for the data collected with 15 ms time interval. The fraction of bound molecules (F_bound_) and the diffusion coefficient of the bound molecules (D_bound_) are presented. **d)** Spot-On two-state kinetic modeling with 15 ms time interval data provides values for F_bound_ and D_bound_, which closely resembles the values obtained using Sojourner. The names of the software packages are mentioned at the top of each panel.

**Fig. 2:**
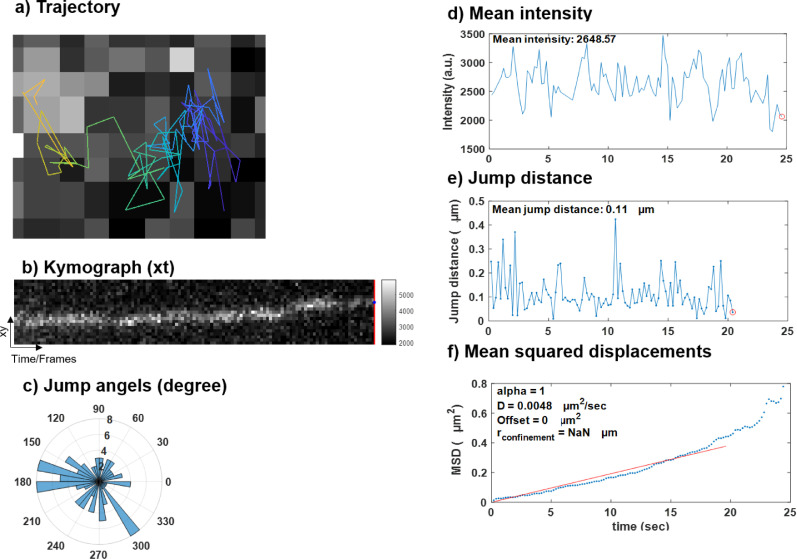
TrackIt-based analysis of individual trajectory: a) TrackIt provides visualization for the entire trajectory of a single particle and it can be color-coded based on time, jump distance, and intensity. Here it is color-coded based on time. b) Visualization of the entire trajectory by kymograph (xt projection). c) Jump angle distribution for one particular track. d) Mean fluorescence intensity for the individual particle/track over time. e) Jump distances for the individual particle/track over time. f) Mean squared displacements of individual particle/track over time.

**Fig. 3:**
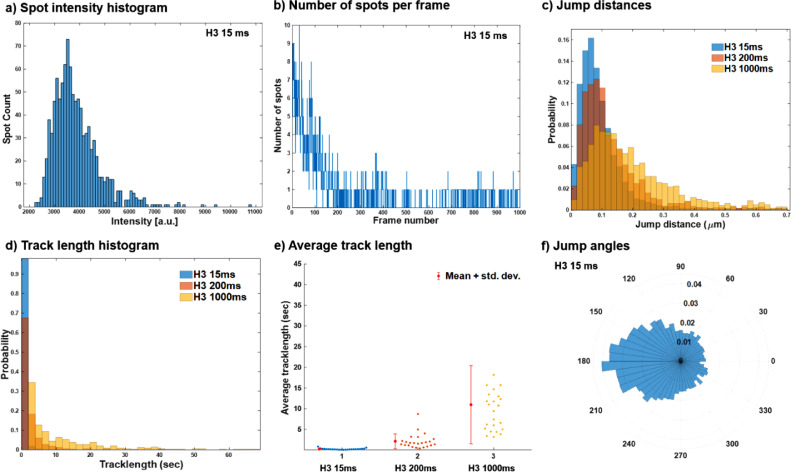
TrackIt-based batch analysis for the entire dataset: a) Spot intensity histogram shows the probability of tracking two molecules as a single molecule. The single peak represents the tracking of single molecules only, however, there are a few spots (negligible in numbers) with a higher intensity that represents two or more molecules at the same place, at the same time. **b)** Number of spots detected per frame. **c)** Jump distance histograms for three datasets. **d)** Track length histogram represents the residence time of the tracks for three datasets. **e)** Average track length represents the mean residence time. Each dot represents the average track length for each movie (total 24 movies). **f)** Jump angle distribution of all the tracks.

The shared data is a collection of time-lapse movies acquired using fast (0 ms interval, 15 ms exposure) and slow (200 and 1000 ms intervals, 15 ms exposure) imaging regimes (Mendeley data, Table 1: https://data.mendeley.com/datasets/xkjp4nm33h) [8]. We tracked these movies using three MATLAB-based software: MatlabTrack, TrackIt, and Diatrack (followed by Sojourner and Spot-On). The tracked files are also available on Mendeley data (Table 1). We have presented the biophysical parameters quantified from this data in Figs. 1, 2, and 3.

**Table 1:** List of files/folders shared through Mendeley data.

Folders	Subfolders
Raw Microscopy Images (.tiff)	Histone H3_15 ms interval_15 ms exposure_1000 frames
	Histone H3_200 ms interval_15 ms exposure_200 frames
	Histone H3_1000 ms interval_15 ms exposure_100 frames

MatlabTrack files (.mat)	Histone H3_15 ms interval
	Histone H3_200 ms interval
	Histone H3_1000 ms interval

TrackIt files (.mat)	Histone H3_15 ms interval
	Histone H3_200 ms interval
	Histone H3_1000 ms interval

DiaTrack files (.mat)	Histone H3_15 ms interval

Sojourner files (.csv)	Histone H3_15 ms interval

## 3 Experimental Design, Materials and Methods

### 3.1 Genetic engineering of yeast strains for single-molecule tracking

For the single-molecule tracking experiment, we made the following genetic manipulations in the yeast genome using PCR-based homologous recombination: 1) deletion of the *PDR5* gene, 2) expression of GFP-LacI-NLS (as a localization marker for nucleus), 3) HHT1-HaloTag. Please refer to [1] for detailed methodology.

### 3.2 Culturing cells for single-molecule imaging

The cells were streaked on the CSM plate from the glycerol stock (stored at -80 °C freezer) and incubated at 30 °C for 48 hr. A single colony was inoculated in 3 ml CSM broth and grown at 30 °C under shaking conditions (230 RPM) for 20-24 hr. 50 µl of this culture was inoculated in fresh 3 ml CSM broth and grown at 30 °C under shaking conditions (230 RPM) for 5-6 hr to bring the cells to the log phase. Cells were pelleted by centrifugation (2000 RPM for 2 min) and resuspended in 1 ml of fresh CSM, JF646-HTL was added at 0.005 nM concentration and kept for shaking for 30 min. Cells were pelleted again by centrifugation (2000 RPM for 2 min) and washed twice with 3 ml of fresh prewarmed CSM media to remove unbound JF646-HTL. Cells were finally resuspended in 20 µl of CSM media. 3 µl of this cell suspension was taken on the LabTekII imaging chamber, covered by a nutrient agarose pad (8 x 8 mm), and observed under the custom-built HILO microscope.

### 3.3 Image acquisition

For acquiring time-lapse movies for single-molecule imaging, we used two different imaging regimes: fast imaging (0 ms interval, 15 ms exposure) and slow imaging (200 or 1000 ms interval, 15 ms exposure). The movies acquired with fast imaging regimes were used to quantify the diffusion parameters (such as mean-squared displacement (MSD), diffusion coefficient (D), the fraction of bound and unbound molecules, and target-search mechanism). The movies acquired with slow imaging regimes were used to quantify the residence time. The time-lapse movies were acquired using a 647 nm laser (power 1 mW), whereas, the reference images were acquired as single timepoint images (after acquiring the movie in a 647 nm channel) using a 488 nm laser (power 100 µW).

### 3.4 Analysis of single-molecule images

Three MATLAB- based tracking software packages (MatlabTrack, TrackIT, DiaTrack (Sojourner and Spot-On)) were used to quantify various biophysical parameters from these time-lapse movies. Please refer to Podh et al. 2022 (STAR Protocols) for detailed methodology. Figs. 1 and 2 represent the various biophysical parameters quantified for histone H3.

## Ethics Statement

This work is the authors' own original work, which has not been previously published elsewhere.

This work did not involve human subjects or animal experiments. The data was not collected from social media platforms.

## CRediT authorship contribution statement

**Nitesh Kumar Podh:** Methodology, Formal analysis, Validation, Visualization, Data curation, Writing – original draft, Writing – review & editing. **Ayan Das:** Methodology, Formal analysis, Validation, Visualization, Data curation, Writing – original draft, Writing – review & editing. **Partha Dey:** Validation, Writing – review & editing. **Sheetal Paliwal:** Validation, Writing – review & editing. **Gunjan Mehta:** Conceptualization, Methodology, Investigation, Resources, Data curation, Visualization, Writing – original draft, Writing – review & editing, Supervision, Project administration, Funding acquisition.

## Declaration of Competing Interest

The authors declare that they have no known competing financial interests or personal relationships that could have appeared to influence the work reported in this paper.

## Data Availability

Single-Molecule Tracking dataset for histone H3 (Hht1) in yeast Saccharomyces cerevisiae (Original data) (Mendeley Data).
